# The Role of Limbal Epithelial Stem Cells in Regulating Corneal (Lymph)angiogenic Privilege and the Micromilieu of the Limbal Niche following UV Exposure

**DOI:** 10.1155/2018/8620172

**Published:** 2018-05-08

**Authors:** M. Notara, A. Lentzsch, M. Coroneo, C. Cursiefen

**Affiliations:** ^1^Department of Ophthalmology, University of Cologne, Cologne, Germany; ^2^Department of Ophthalmology, University of New South Wales, Prince of Wales Hospital, Sydney, Australia; ^3^Ophthalmic Surgeons, Sydney, Australia; ^4^East Sydney Private Hospital, Sydney, Australia; ^5^Look for Life Foundation, Sydney, Australia; ^6^Center for Molecular Medicine Cologne (CMMC), University of Cologne, Cologne, Germany

## Abstract

The cornea is a clear structure, void of blood, and lymphatic vessels, functioning as our window to the world. Limbal epithelial stem cells, occupying the area between avascular cornea and vascularized conjunctiva, have been implicated in tissue border maintenance, preventing conjunctivalisation and propagation of blood and lymphatic vessels into the cornea. Defects in limbal epithelial stem cells are linked to corneal neovascularisation, including lymphangiogenesis, chronic inflammation, conjunctivalisation, epithelial abnormalities including the presence of goblet cells, breaks in Bowman's membrane, persistent epithelial defects and ulceration, ocular surface squamous neoplasia, lipid keratopathy, pain, discomfort, and compromised vision. It has been postulated that pterygium is an example of focal limbal deficiency. Previous reports showing changes occurring in limbal epithelium during pterygium pathogenesis suggest that there is a link to stem cell damage. In this light, pterygium can serve as a model disease of UV-induced stem cell damage also characterised by corneal blood and lymphangiogenesis. This review focuses on the role of corneal and limbal epithelial cells and the stem cell niche in maintaining corneal avascularity and corneal immune privilege and how this may be deregulated following UV exposure. We present an overview of the PUBMED literature in the field as well as recent work from our laboratories.

## 1. Introduction

The cornea is the avascular, clear outer tissue of the ocular surface with important refractive and barrier functions. The cornea consists of 5 layers: epithelium, Bowman's layer, stroma, Descemet's membrane, and endothelium [1].

**Figure 1:**
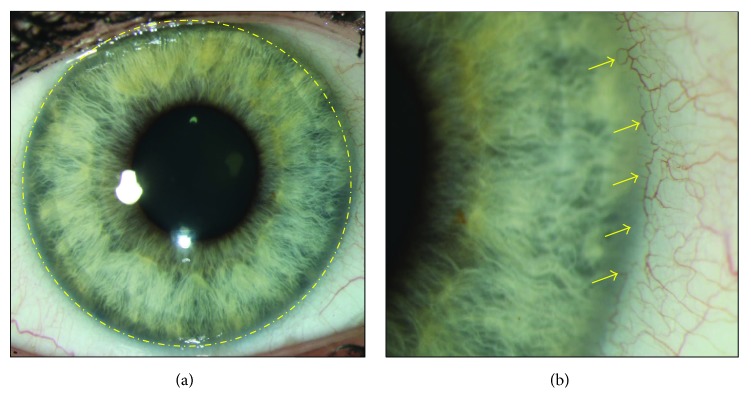
(a) Location of the limbus, the barrier between physiologically hem- and lymph-vascularized conjunctiva and avascular and transparent cornea. Limbal stem cells reside in the limbal region of the ocular surface, along the dotted line. (b) The arrows highlight the vascular plexus which is visible in the conjunctiva and stops at the limbus.

The corneal epithelium is the outermost layer of the cornea and is bathed by the tear film. It comprises 5-6 layers of stratified nonkeratinized epithelium with a total thickness of 40–50 *μ*m. The most superficial epithelial cells have microvilli and microplicae that connect them to the mucinous layer of the tear film. Intercellular tight junctions form a barrier to pathogens and fluid. Basal epithelial cells that are able to undergo mitosis form the innermost layer of cells. Epithelial stem cells are found in the limbal basal epithelium [1] in the limbus, the transition area separating the cornea and the conjunctiva and underlying sclera (Figure 1(a)) [2]. Infoldings in the limbus, which greatly increase its surface area, are known as the palisades of Vogt. These palisades are enmeshed in a vascular plexus which provides additional nutrition to that obtained from the aqueous humour and the tear film [2] (Figure 1(b)).

Desmosomes hold cells together while hemi-desmosomes anchor the basal epithelial cells to the basal lamina. The underlying acellular Bowman layer is a condensate of fine, randomly arranged collagen fibrils [3]. The stroma comprises 90% of the corneal thickness. Due to its biomechanical structure, mostly a precise organization of dense collagen fibrils (collagens I and V) regularly arranged in lamellae, it provides transparency and mechanical strength. Collagen fibrils are synthesized by keratocytes, the main cell type occupying the stroma, mostly residing in the anterior stroma [4]. In more recent years, corneal stromal stem cells have been identified in the anterior limbal stroma in close proximity to epithelium stem cells [5].

Under the corneal stroma is Descemet's membrane, a thick acellular basement membrane consisting of collagen IV. The collagen is secreted by endothelial cells and anchors the endothelium to the stroma. The endothelium is a monolayer of squamous cells arranged in a hexagonal pattern. These cells keep the cornea dehydrated by pumping fluid out of the cornea via an osmotic gradient from the hypo-osmotic stroma to the isotonic aqueous humour. While central endothelial cells appear to be quiescent and endothelial cell density declines throughout life from 3500 cells/mm at birth to 2600 cells/mm in elderly, there is increasing evidence of endothelial progenitor cells, located in the Schwalbe's line region [6].

A wide range of diseases can afflict any or all of these corneal layers and may ultimately lead to compromising of corneal transparency and thus corneal blindness. Here, we will focus on the role of the external limbus and especially the residing stem cells in forming a barrier against neovascularisation.

## 2. Limbal Epithelial Stem Cell Characteristics and Division

Corneal epithelium integrity is essential for corneal transparency and refraction. This epithelial layer is regenerated by a limbal epithelial stem cell (LESC) population located in the basal cell compartment of the limbus.

A typical characteristic of limbal stem cells is that they are mitotically inactive [7, 8] while they have a high proliferative potential which is manifested during the transient amplifying stage [9]. Basal limbal epithelial cells have a slow cycle which is shown by the retaining of the marker triturated thymidine (BrdU) for long chasing periods [10]. Within the limbal basal cell population, the putative LESC are cuboidal in shape and have a smaller volume compared to the basal cells of the central and peripheral cornea [11]. They have a high nucleus to cytoplasm ratio and nuclei rich in heterochromatin with no well-defined nucleoli [11, 12]. Clonal analysis of cells from the olimbal compartment confirmed that they have a higher proliferative capacity compared to their counterparts derived from the central or peripheral corneal regions [13]. Stromal crypt structures corresponding to putative stem cell niches facilitate the crosstalk of limbal basal cells with the neighbouring vessel network, extracellular matrix, and other cell types [11, 14, 15]. According to recent reports, the limbal basal cells contain melanin, which prevents UV damage, while melanocytes found in close proximity play a supporting role in preserving stemness [10, 16].

Putative stem cells residing in the basal limbal epithelium are integrin alpha9 positive and connexin 43, E-cadherin, nestin, involucrin, K12, and K3 negative, with higher expression of EGFR [11]. Although a LESC-specific marker remains unknown, certain proteins, including P63a and especially its ΔNP63*α* isoform [17], ABCG2 [18], cytokeratin15 [19], cytokeratin 14 [20], cytokeratin 7 [21], frizzled 7 [22], and more recently ABCB5 [23], are most commonly used as putative stem cell markers for these cells. Due to the lack of a specific marker, however, a panel of the aforementioned markers should be used to optimally characterise putative LESCs.

In order to maintain the stem cell population, stem cells are thought to divide asymmetrically to produce one transient amplifying (TA) cell and one stem cell [24]. Markers of TA cells in the limbus include cytokeratin 19 [25] and endolase-alpha [11]. Although some data suggest that asymmetrical division occurs across the entire corneal epithelium, it is reported that asymmetrical cell division in adults occurs exclusively in the stem cell containing limbal epithelium, as suggested by the expression patterns of some molecules which drive cell stratification and differentiation [26]. The TA cells proliferate quickly deriving terminally differentiated cells which can maintain the corneal epithelium. Notably, there is evidence that mammalian stem cells may also divide symmetrically [27]. In symmetric stem cell division, a stem cell gives rise to two identical daughter cells—either two stem cells or two TA cells [28].

### 2.1. The Limbal Epithelial Stem Cell Niche

**Figure 2:**
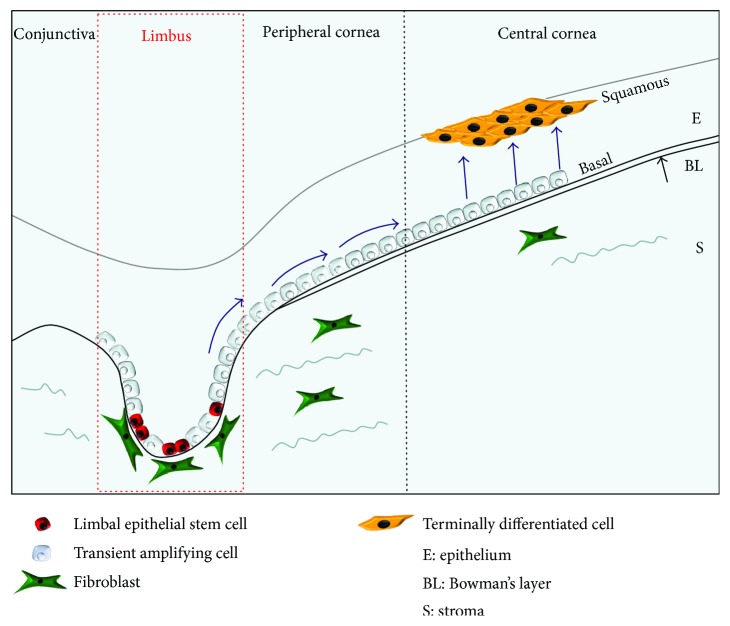
The limbal epithelial stem cells (found in the basal limbal epithelium) divide to produce transient amplifying cells which migrate towards the apical layers of the corneal epithelium and eventually become terminally differentiated [51].

LESC are believed to reside in the basal layer of the limbal region of the cornea. The nonuniform intersection between the limbal epithelium and stroma provides shelter from shear forces while the adjacent blood vessels provide a source of nutrition for the niche cells [29]. While the limbal stem cells that reside are normally quiescent upon injury or due to normal wear and tear of the corneal epithelium, they enter the TA state while migrating to the site where they are needed (Figure 2).

The limbal palisades of Vogt have been proposed as the site of the LESC niche [30]. Clinically, these can be examined using a slit-lamp microscope and look like radial linear structures measuring up to 1 mm in length [31, 32]. Histological, photomicrographic, and angiographic studies have shown that the palisades are fibrovascular and that there are “ridges of thickened epithelium” in the interpalisade section [31, 32]. Dua et al. [33] identified the limbal epithelial crypt, a novel anatomical structure extending from the palisades of Vogt and is proposed as a LESC stem cell niche. Cytokeratin 14 immunopositivity demonstrated the epithelial nature of the crypt cells, while ABCG2 expression suggested that the crypts may contain putative stem cells [33].

An early suggestion of the existence of limbal stem cells was provided by Mann during the 1940's. Using both laboratory investigations and clinical observations, she documented melanin shift from the limbus to towards an epithelial defect during corneal wound healing [34] Davanger and Everson in 1971, using similar observations, proposed that “the limbal papillary structure serves as a generative organ for corneal epithelial cells.” They also proposed that “a failure in the limbal structure may be the cause of pterygium” [30].

Since then, further evidence was reported to back the theory that the stem cells reside in the limbus. This evidence includes the following: the limbal basal cells have a much greater proliferative capacity compared to corneal epithelial cells from the centre and the periphery [13]; limbal epithelial basal cells retain BrdU labelling thus indicating that they are slow cycling [10]; and wounding or surgical removal of the limbus results in delayed healing and conjunctivalisation of the cornea [35, 36].

Despite recent controversy regarding the presence of corneal stem cells in the central cornea as well as the limbus [37, 38], most experimental and clinical evidence leads to the conclusion that the limbus remains the foremost LESC niche structure. LESCs are considered to be unevenly distributed throughout the human limbus, being more abundant in the superior and inferior regions compared to the temporal and nasal [39, 40]. Interestingly, infolding can greatly increase the surface area available by as much as two orders of magnitude as compared to a flat surface [41] and this has been recognized in attempts to create an artificial limbal structure [42]. The physical size as well as the colony forming efficiency of cells derived from these LESC-rich areas is reduced with age [43].

Adult stem cells have innate properties that define their behaviour, but they also depend on specific environmental niches in which they are kept undifferentiated and quiescent and their progeny is guided to a predefined cell fate. Further understanding of the mechanisms that control these SC-niche interactions is necessary to understand normal SC function and tissue homeostasis. The composition of the extracellular matrix of the niche is considered a key component as it has been shown that the basement membrane composition of the limbus is significantly different compared to the one of the cornea [44–46] especially in the distribution of collagens [46], laminins [47], tenascin [25], and integrins [48].

### 2.2. Limbal Epithelial Stem Cell Deficiency and Corneal Neovascularisation

The limbal epithelial stem cells are important for epithelial cell renewal and closure of wound defects. Corneal epithelial cells have a lifespan of 7–10 days [49]. If there is a defect in the corneal barrier, a nonmitotic wound healing is perceived by migration and cell spreading of healthy cells at the border of the defect, followed by mitotic cell renewal to reconstitute tissue homeostasis [50].

A dysfunction or depletion of LESC in combination with destruction of their stem cell niche may result in a limbal stem cell deficiency (LSCD) that significantly alters tissue homeostasis. Conditions leading to LSCD may be congenital (as in aniridia), or acquired such as in chemical and thermal burns, inflammatory eye disease (e.g., ocular cicatricial pemphigoid or Stevens-Johnson syndrome), and contact lens-related hypoxia [51].

In LSCD, a crucial alteration in tissue homeostasis occurs due to a direct depletion of the LESCs (in chemical injuries and burns), their absence (in aniridia), or due to a persistent tissue inflammation caused by an imbalance between pro- and antiangiogenic factors, changing the structural integrity of the corneal surface. Thus, corneal transparency and visual function are no longer maintained, and as a result, corneal neovascularisation with recurrent epithelial defects, corneal scarring, corneal conjunctivalisation, and formation of a fibrovascular pannus occurs. The diagnosis of LSCD remains on typical clinical manifestations on examination. Symptoms of LSCD often include impaired vision, discomfort, redness, tearing, and photophobia [52].

A characteristic finding on slit-lamp examination for LSCD is corneal conjunctivalisation due to a migration of conjunctival epithelium and blood vessels when the barrier function of the limbus is lost. Other manifestations comprise an irregular epithelium, recurrent epithelial defects that stain with fluorescein, unstable tear film, neovascularisation and keratinization of the corneal surface, scarring, calcification, pannus formation, and loss of corneal transparency [53, 54].

To depict the severity of the disease, it is important to outline the extent of deficiency (partial or total cornea affected) and to evaluate whether only one or both eyes are affected. Sectorial ingrowth of conjunctival epithelium occurs in partial LSCD because intact limbal epithelial stem cells lie within the areas of LSCD. In total LSCD, a complete conjunctivalisation of the corneal surface is seen and, in severe cases, corneal melting and perforation may result. So far, there is no consisting grading system for severity of LSCD [52]. Clinical diagnosis can be supplemented by impression cytology and immunohistochemistry for cytokeratins K3 [55] and K13 [56], impression cytology for goblet cells, or in vivo confocal biomicroscopy [57]. An intimate regulation of epithelial cell proliferation is the key to maintain corneal avascularity. Animal models of increased epithelial proliferation such as in Destrin-mutant CORN1 mice also show spontaneous corneal neovascularization [58, 59].

## 3. Angiogenic Privilege and Regulation of Pro- and Antiangiogenic Signals in the Cornea and Limbus

The corneal tissue produces factors that act as a barrier to blood and lymphatic endothelial vessels in order to maintain transparency and vision. Specifically, the corneal epithelial basement membrane plays a fundamental role in the production of antiangiogenic factors. Strong antiangiogenic molecules including endostatin are derived from an extracellular matrix part of the EBM, namely, collagen type XVIII [60]. Endostatin opposes endothelial cell proliferation by halting their cell cycle to G1 while inhibiting the eliumelial growth factor- (VEGF-) induced tyrosine phosphorylation of KDR/Flk-1 and activation of p38, MAPK, ERK, and p125FAK, which are downstream events of KDR/Flk-1 signalling and regulate VEGF-induced proliferation and migration in vascular endothelial cells [61]. In addition, the angio-inhibitory thrombospondin and tissue inhibitor of metalloproteinase-3 are bound in the EBM. Thrombospondins (TSP) are produced by a family of five genes encoding glycoproteins which control a variety of functions related to the extracellular matrix. TSP-1 and TSP-2 are classified as a subfamily with potent antiangiogenic effects [62, 63]. Both proteins are expressed in the cornea and contribute to its avascularity [64]. TSP-1 is a multifunctional extracellular matrix protein and inhibitor angiogenesis inhibitor which functions by binding to transforming growth factor TGF*β* and thus promoting its activation. It may also hinder angiogenesis by halting endothelial cell viability and migration [65] through triggering vascular endothelial cell apoptosis mediated by CD36 [66] as well as through binding and thus affecting the availability of growth factors essential for endothelial cell growth and functionality such as heparan sulfate proteoglycans [63]. Immunoreactivity for TSP-1 was observed in human and bovine corneal endothelium, epithelial basement membrane, and posterior Descemet's membrane by light microscopy [64]. Most recently, our group could gain a deeper insight into the molecular mechanisms of the antilymphangiogenic effect of TSP-1 [67]. It was shown that TSP-1 knockout mice show the phenotype of the autoimmune disease Sjögren' s syndrome at approximately 6 months of age and are used as a model for dry eye disease (DED) [68]. The phenotype is consistent to DED as it exhibits persistent corneal epithelial defects, histologically detected immune cell infiltrates in the lacrimal gland, and ingrowth of lymphatic vessels into the central cornea which emerge due to the absence of immunoregulatory effect of TSP-1. We have demonstrated that TSP-1 expressed the TGF*β*-induced expression of the prolymphangiogenic VEGF-C and VEGF-D [69, 70] by macrophages through binding to CD36 which is available on their membranes [67]. The absence of TSP-1 leads to accumulation of VEGF-C and VEGF-D over time thus tipping the balance towards a prolymphangiogenic microenvironment. Additionally, the lack of TSP-1 induces the upregulation of the proinflammatory cytokine MCP-1 (monocyte chemotactic protein-1) which explains the presence of more VEGF-C producing macrophages in the cornea of TSP-1-deficient mice [67]. It is therefore demonstrated that the loss of TSP-1 contributes in many ways towards corneal lymphangiogenesis.

TSP-2 lacks a TGF*β*-binding site, so it acts antiangiogenically mainly by driving endothelial cells to cell cycle arrest while not causing their apoptosis [62]. Damaged corneas due to both infections of injury show lower levels of TSP2 demonstrating its crucial role in maintaining corneal antiangiogenic balance [71–73].

### 3.1. VEGF Signalling and the Antiangiogenic Decoy Mechanisms of the Limbus and Cornea

Corneal cells largely contribute to the antiangiogenic balance in the cornea. Early reports suggest that corneal epithelium acts antiangiogenically [74–76], as the different cell populations of the cornea collectively promote the antiangiogenic balance leading to corneal avascularity. Although the exact mechanisms are not yet fully elucidated, the cells of the limbus act as a physical and physiological barrier against blood and lymphangiogenesis [73, 77]. The resident epithelial stem cells in the limbus constantly replenish the cornea with epithelial cells which are lost due to normal wear and tear or injury [15]. Other studies demonstrated that corneal fibroblast signalling permits vessel invasion under pathological conditions [78–80] while our own data illustrated the proangiogenic paracrine activity of human limbal fibroblasts on blood and lymphatic endothelial cells by promoting migration, proliferation, and tube formation compared to human limbal epithelial cells and controls [81].

Corneal neovascularisation is regulated by the VEGF family of proteins which includes VEGF-A, VEGF-B, VEGF-C, and VEGF-D, placenta growth factor (PlGF), and the viral VEGF homologue VEGF-E. VEGF-A enables hemangiogenesis and vascular permeability. VEGF-B facilitates nonangiogenic tumour progression [82, 83]. Four different VEGF-A isoforms derived from alternative mRNA splicing, namely, VEGF121, VEGF165, VEGF189, and VEGF206, have been identified in humans [84]. VEGF189 and VEGF206 are bound on the extracellular matrix while the secreted VEGF121 and VEGF 165 are shorter [84]. VEGF-A binds to two high-affinity receptor tyrosine kinases, VEGFR-1 (FMS-like tyrosine kinase-1 or Flt-1) and VEGFR-2 (kinase insert domain-receptor or KDR), which are abundant in vascular endothelial cells. VEGF-C and VEGF-D on the other hand bind to VEGFR-3 (or FMS-like tyrosine kinase-4, Flt-4) and are mainly found in lymphatic endothelial cells [85, 86]. Van Setten was the first to describe the presence of VEGF in the cornea by immunolocalisation in the basal layer of corneal epithelial cells [87]. The first report to link corneal neovascularisation following limbal epithelial deficiency to VEGF induction and inflammation was carried out using a rat model [88]. Corneal neovascularisation occurs following the infiltration of leucocytes which secrete VEGF [89, 90]. Specifically, following corneal cautery, VEGF165 and VEGF189 mRNA were induced while VEGF mRNA expression was detected in neutrophils and macrophages by in situ hybridisation [89]. VEGFs as well as TGF*α* and TGF*β*1 were also localised in basal corneal epithelium as well as in endothelial cells of formed vessels and infiltrating immune cells (T-lymphocytes, macrophages) [91]. More recently, IFN-*γ*-secreting natural killer (NK) cells emerged as a new player in corneal by inducing upregulation of VEGF expression by macrophages. NK cell depletion in a transgenic model reduced macrophage numbers in the cornea as well as mRNA expression of VEGF-A, VEGF-C, and VEGFR3 while in a laser-induced corneal neovascularisation model, NK cell depletion leads to a reduction of neovascularisation while significantly downregulating IFN-*γ* and VEGFs in the choroid [92].

New therapies target both lymphatic and blood vessel formation in the cornea as they also contribute to corneal graft and limbal stem cell graft rejection and sensitization in prevascularized corneas [93]. Corneal lymphangiogenesis is mainly regulated by VEGF-C which promotes growth of both lymphatic and blood vessels. However, VEGF-A also is stimulating both lymph- and hemangiogenesis via macrophage recruitment [70] while its early postoperative targeting led to reduction of both types of vascularisation and improved corneal transplantation outcome [94]. Under physiological conditions, VEGF-C is expressed in conjunctiva but not in the cornea; however, corneal injury in a rat model induces mRNA upregulation of VEGF-C consistent with corneal hem- and lymphangiogenesis [95]. Both vascular endothelial and perivascular cells were confirmed to express VEGF-C which promoted both lymphangiogenesis and hemangiogenesis mediated by FGF-2 [96]. Lymphatic vessel endothelial cells express specific proteins including podoplanin and the lymphatic vessel endothelial hyaluronan receptor (LYVE-1) [97]. Generally, the corneal lymphangiogenesis is proportional to the extent of corneal hemangiogenesis possibly due to the chemotactic action of VEGF-A which attracts macrophages as well as monocytes which subsequently secrete prolymphangiogenic VEGF-C and VEGF-D thus amplifying both types of neovascularisation. This effect is reversed by suppression of macrophages [94].

The corneal epithelium deploys a number of receptor decoy mechanisms to neutralise potential proangiogenic signals especially VEGF molecules. Via these mechanisms, an intact epithelium contributes to the clinically observed corneal avascularity. Cursiefen and colleagues demonstrated that VEGFR-3 is ectopically expressed in the corneal epithelium [98]. VEGFR-3 binds to VEGF-C and VEGF-D, which are the major regulators of lymphangiogenesis. When this receptor is locally inactivated in a mouse animal model, there is invasion of lymphatic vessels in the cornea leading to the conclusion that VEGFR-3 acts as a “sink” for its ligands to prevent corneal vascularisation [98]. VEGFR-2, another vascular growth factor receptor, is expressed in the epithelium as a soluble protein, namely, sVEGFR-2 [99]. This protein regulates the progression of lymphatic vessels as demonstrated in a transgenic sVEGFR-2-deficient mouse model which exhibited lymphatic vessel development in the central cornea [100].

Additionally, a soluble VEGF-A receptor, sVEGFR1, expressed in the cornea functions as a decoy receptor for secreted VEGF while neutralising VEGF-A receptors 1 and 2 by heterodimerisation [101–103].

## 4. Effects of UV Irradiation to the Limbal Niche: Pterygium as an UV-Induced Disease Model

**Figure 3:**
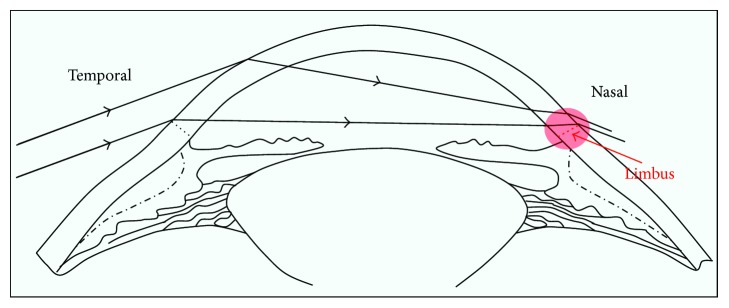
Schematic representation of the peripheral light focusing effect, occurring on the nasal side of the limbus. This site is associated with increased incidence of pinguecula and pterygium, conjunctival tumours which are associated with UV damage [106].

UV radiation is injurious to various ocular structures and may lead to partial or total blindness [104, 105]. The cornea is quite susceptible to UV irradiation owing to its transparency as well as to its curved shape which is contributing to a peripheral light focusing effect by which the UV irradiation is intensified 20-fold at the nasal limbus [106, 107] (Figure 3). This is the site where pterygium, a noncancerous often bilateral vascularized growth of the cornea, occurs. The pterygium invades the limbal barrier which separates the cornea from the conjunctiva and encroaches the superficial cornea often covering the visual axis. It is characterised by the growth of a wing of altered squamous epithelium, a stroma of activated proliferating fibroblasts, goblet cell hyperplasia, neovascularization, inflammation, and altered extracellular matrix.

Vision can be impaired by a number of different mechanisms [104, 108], including induced astigmatism, invasion of the visual axis, visual field loss, diplopia, a dry eye state, and contact lens intolerance [109].

One possible mechanism of UV damage is via an effect of peripheral light focusing on corneal nerves as they traverse the limbus [110]. Release of neuropeptides such as substance P, acting via NK1 receptors, could act as a chemoattractant for fibroblast and vascular endothelial cells [110].

There is strong histochemical evidence that pterygium onset is accompanied by coexpression of matrix metalloproteinases (MMPs) [111, 112] and basal limbal markers in limbal epithelial cells thus suggesting that the condition may indeed be a limbal stem cell disorder [104]. The specific effect of chronic UV irradiation to the phenotype of limbal stem cells as well as their direct involvement in the onset and development of the disease is not fully elucidated. It is sensible to suggest that since stem cells have a slow cell-cycle and persist through most of the lifetime of an individual, they can accumulate UV-induced damage over time which can cause benign or malignant transformation. At the same time, UV damage of other cell types residing in the LESC niche, including limbal fibroblasts, may compromise the stem cell phenotype and functionality. We have provided evidence that perhaps the earliest evidence of UV damage may be in vivo evidence of corneal invasion of Fuchs' fleck-like lesions at the head of primary and recurrent pterygia, pinguecula, and even in clinically normal nasal and temporal limbus in sun-damaged individuals [113].

### 4.1. Immune Cells and Pterygium

Immune cell subpopulations are normally located in small numbers on the ocular surface. These cells, mainly dendritic cells (DCs), abundant in conjunctiva but also present in the cornea, are believed to act as sentinels to external signals and insults [114]. The location of the cells facilitates a direct association owing to spatial proximity between nerves and resident immune cells in the cornea suggesting signalling crosstalk between the two structures [115].

To describe the stratification of antigen-presenting cells of the cornea, Knickelbein et al. utilized transgenic mice GFP-reporting for CD11c in conjunction with immunohistochemical staining. pCD11c(+) dendritic cells (DCs) are present in the basal epithelium, evidently embedded in the basement membrane. pCD11c(−) CD11b(+) putative macrophages which weakly expressed MHC class II were located beneath the DCs and adjacent to the stromal side of the basement membrane. Finally, MHC class II(−) pCD11c(−) CD11b(+) cells consist of a network through the remainder of the stroma [116].

DCs of the limbal stroma exhibit a distinct phenotype compared to their corneal counterparts. Specifically, they were found to express A8 and A9 which are subtypes of S100 proteins. These cells were positive for CD45, HLA-DR, and CD11c, which are characteristic markers of DCs. Thus, expression of A8 and A9 may help to distinguish between subpopulations of DCs which are present in different regions of the cornea and may impact on their maturation state [117].

In pterygium, the numbers of resident DCs are significantly elevated compared to normal cornea, as confirmed by utilising an in vivo laser scanning confocal microscope where they appear as highly reflective cellular structures with characteristic dendrites [118–120]. The increased numbers of Langerhans cells in pterygium may suggest an elevated antigenic and proliferative activity in the conjunctiva and is linked to increased vimentin expression by epithelioid cells [121].

The various immune cell populations in the cornea, as they react to UV irradiation, may affect developments regulating pterygium pathogenesis. Our own observations suggest that UVB irradiation correlates to an increase of immune cell-recruiting cytokines thus amplifying immune cell numbers and inflammation [122].

Focal regulation of the ocular surface immune system may also be involved in pterygium pathogenesis [104, 106, 123, 124]. Langerhans' cells are found among corneal epithelial cells, but they are more numerous at the limbus and in the conjunctiva [125]. In experimental models, exposure of the skin to UV radiation has been shown to elicit suppressor T lymphocytes, inducing immune tolerance. This immune tolerance, in turn, has been linked to a reduction in the number of Langerhans cells, morphological changes, and failure to present antigens to T lymphocytes [126]. It is therefore possible that a similar mechanism regulating tolerance may exist in the cornea.

It has also recently been shown that the rate of rejection of mouse heterotopic corneal allografts is reduced after in vitro pretreatment with UV light and that this reduction is related to a reduction or alteration of Langerhans cells which reside in the ocular surface [126, 127]. Localised UV irradiation by albedo concentration at the medial limbus may thus induce immune tolerance, with the result that conjunctival cells may no longer recognize the junction between cornea and conjunctiva and invade the cornea at this site, thus causing pterygium [123]. Despite recent advances in understanding the function of immune cell populations in the cornea, the exact mechanism by which the balance is tipped towards reduced tolerance inflammation remains elusive.

### 4.2. UV-Induced Dysregulation of the Limbal Niche and Subsequent Inflammation and Neovascularisation Events

UV exposure can induce extensive alterations linked to pterygium pathogenesis. Specific alterations occurring in the epithelial compartment of the limbus during the disease, such as MMP expression by basal epithelial cells [104], suggest that there is a link to stem cell damage. In this light, pterygium can serve as a model disease of UV-induced stem cell damage also characterised by corneal blood and lymphangiogenesis [128].

Signs of both direct and indirect DNA UV damage have been detected in pterygium either by formation of base dimers following the direct UV absorption by DNA or indirectly via by-products of oxidative stress. For instance, pyrimidine dimers, which are molecular lesions formed by thymine and cytosine dimers produced by photochemical reactions [129], have been immunolocalised in pterygium and recurrent pterygium tissue along with p53 activation, thus demonstrating the occurrence of direct DNA damage and activation of DNA repair mechanisms [130]. In case of indirect damage, UV irradiation is inducing the formation of reactive oxygen species which cause oxidative stress [131]. This is manifested by cellular lipid and membrane injury as well as by DNA damage [132]. 8-Hydroxydeoxyguanosine (8-OHdG), a prominent marker for oxidative damage due to its high mutagenic effect [133, 134], has been detected in human pterygium specimens [135].

UV-induced changes in the cornea are characterised by the upregulation of proinflammatory cytokines including interleukin- (IL-) 1 [136], IL-6 [137], IL-8 [137], and tumour necrosis factor alpha (TNF*α*) [138] which correspond to the increased numbers of inflammatory cells found in pterygium specimens. In addition, growth factors including vascular endothelial growth factor (VEGF) [139, 140], platelet-derived growth factor (PDGF) [141], transforming growth factor beta (TGF*β*), and matrix metalloproteinases (MMP) [112, 142, 143]—especially MMP1—are upregulated. The prolymphangiogenic VEGF-C [98] and its receptor VEGFR-3 are also upregulated in pterygium specimens [139]. This increase may provide an explanation for the increased density of lymphatic vessels linked to pterygium recurrence and staging [128, 144].

Collectively, changes in the above factors mediate UV-induced inflammation, neovascularisation, hyperplasia, and tissue remodelling associated with pterygium and have been observed post UV radiation in the normal cornea, conjunctiva, and pterygium tissue as well as in ex vivo cultivated cells [137, 145]. Given these findings, it is important to identify whether this damage is occurring to the LESCs and their supporting limbal fibroblasts and if protecting the niche from the UV can effectively prevent it.

Our group has recently used an *in vitro* approach to study UVA- and UVB-induced changes in human limbal epithelial cell and fibroblast phenotype and functionality and their paracrine signalling regulating (lymph)angiogenesis and inflammation [81, 122].

We used primary human limbal epithelial cells and fibroblasts which were received 5.2 J/cm^2^ of UVA or 0.02 J/cm^2^ of UVB irradiation. Our findings suggested that this short-term UV irradiation induced the loss of putative stem cell character of limbal epithelial cells, as their putative LESC marker expression and colony-forming efficiency were significantly decreased. Notably, limbal epithelial cells which were cocultured with UV-irradiated limbal fibroblasts also lost their putative LESC characteristics. For the first time, we showed that UVA and UVB disrupted the function of limbal fibroblasts in maintaining the limbal epithelial cell phenotype in a coculture [81, 122]. We have previously referred to the significance of the spatial proximity and cell-cell contact between the limbal fibroblasts and putative LESCs for the stem cell maintenance [14]. The epithelial-fibroblast interaction within the niche is the key as the fibroblasts and other niche cells (including melanocytes) promote mechanisms which inhibit stem cell differentiation, including the BMP/Wnt [146], TGF*β*/BMP [147, 148], and Notch [149, 150] pathways. Consequently, a change in the function of limbal fibroblasts, an essential cellular element of the limbal microenvironment, is injurious for the niche regulation.

In terms of paracrine activity following irradiation treatment, while conditioned media from non-UVB irradiated limbal epithelial cells hindered lymphatic endothelial cell tube formation and proliferation, this effect was reversed after the cells were treated with UVB [122]. Contrary, proinflammatory and macrophage-attracting factors including MCP1, TNF*α*, and IFN-*γ* significantly increased as a result of UVB irradiation of limbal fibroblasts. TNF*α* is an important proinflammatory cytokine [151] which causes leukocyte recruitment, oedema, and vasodilatation thus leading to cornea neovascularisation [152]. IFN-*γ* is associated to neovascularisation induced by upregulation of macrophage-produced VEGF [92, 153] while MCP1 acts by promoting angiogenesis [154] as well as a monocyte-attracting chemokine [155]. These data showed that limbal epithelial cells and fibroblasts may contribute to the inflammatory mechanisms occuring in the cornea after UVB irradiation by producing these cytokines.

UVA induced a different response to the paracrine function of limbal epithelial cells and fibroblasts compared to UVB. Specifically, conditioned media produced by both irradiated cell types hindered tube formation and proliferation of lymphatic endothelial cells. MCP1 was downregulated as a result of UVA irradiation of both cell types, while IFN-*γ* was upregulated in limbal epithelial cells [81].

**Figure 4:**
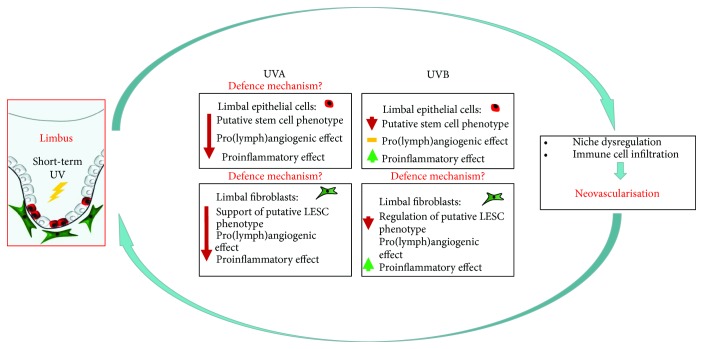
Schematic representation of the impact of UVA and UVB exposure on the limbal epithelial niche: while short-term UVB irradiated limbal fibroblasts downregulate their prolymphangiogenic cytokines, they are no longer able to support the limbal epithelial stem cell phenotype while also causing inflammation. This way, the niche is disrupted and the proinflammatory shift causes the invasion of neutrophils and macrophages promoting neovascularisation (a). On the other hand, short-term UVA also causes a downregulation of proinflammatory and macrophage-attracting cytokines by the cells of the limbal niche. This model put forward the key function of limbal epithelial cells and fibroblasts following short-term UV exposure by employing a defence machinery against proinflammatory and pro(lymph)angiogenic events (b). Schematic picture based on [81, 122].

The results of these studies suggest that limbal epithelial cells and fibroblasts have a double response following short-term UVA and UVB irradiation which is summarized in Figure 4. First, UVB-treated limbal fibroblasts reduce their proangiogenic effect by downregulating factors including VEGF-A and VEGF-C. Possibly, the niche cells employ a defence mechanism to hinder UVB-induced corneal neovascularisation. However, the same cells produce proinflammatory and neutrophil-attracting factors thus enabling tissue repair in addition to inducing a proinflammatory shift that allows corneal neovascularisation via infiltration of immune cells which secrete proangiogenic cytokines [70, 154]. This proinflammatory activity, combined with the loss of the putative limbal stem cell phenotype, may lead to a UVB-induced pro(lymph)angiogenic conditions in the limbus (Figure 4(a)). It is previously reported that macrophage infiltration may lead to induction of corneal neovascularisation [98, 156]. UVB exposure in long term may therefore promote inflammation and hem- and lymphangiogenesis both associated with pterygium progression and recurrence. These results put forward the alterations that short-term UVB treatment may cause to the limbal stem cell niche cellular function and phenotypical characteristics.

Moreover, it is possible that limbal niche cells have a double reaction to short-term UVA exposure. First, limbal epithelial cells and more so fibroblasts hinder their prolymphangiogenic action by decreasing the secretion of cytokines including VEGF-A and VEGF-C. Like in response to UVB, the limbal cells may activate a defence mechanism during the “acute phase,” in order to halt hem- and lymphangiogenesis. Simultaneously, these cells downregulate proinflammatory cytokines to facilitate tissue repair as well as to reduce the inflammatory conditions that enable neovascularisation via infiltration of immune cells which have been shown to secrete proangiogenic factors [70, 154]. The downregulation of MCP-1 in particular may hinder macrophage recruitment which plays an essential role in the amplification of immune cascades and causes VEGF-A, VEGF-C, and VEGF-D-mediated neovascularisation [70, 157]. Taken together, changes following UVA exposure in the putative limbal epithelial stem cell characteristics as well as in the supporting role of limbal fibroblasts may compromise the limbal barrier and eventually the corneal epithelial homeostasis promoting prolymphangiogenic conditions in the limbal region. In this light, long-term UVA insult may contribute to neovascularisation. Possible damage due to long-term UVA exposure is the subject of future studies and not reported in this review, (lymph)angiogenesis via downregulation of macrophage-recruiting cytokines [81]. These data put forward the UVA-induced damage to the limbal niche phenotype and function while showing an antilymphangiogenic and anti-inflammatory effect. If it is possible to ensure the protection of the limbus and its resident stem cells, the therapeutical use of UVA to reduce corneal neovascularisation should be further investigated.

In that respect, the use of UV protection including sunglasses and UV-blocking clear lenses and contact lenses may be a necessary prophylaxis against these damaging effects [158]. UV-blocking contact lenses (UVBCL) especially have been proven preventative against acute photokeratitis caused by UVR overdoses in animal models [159–161] and were shown to be well tolerated in human subjects [162]. Moreover, it has been reported that UVBCL may limit the damage caused by peripheral light focusing effect [163]. Taken together with our recent data describing UV-induced damage on the cells of the limbal niche, the use of UV-protecting measures for the ocular surface is essential for both pterygium patients as well as healthy individuals.

## 5. Conclusions

Even though significant advances have been achieved in unravelling the mechanisms by which the cornea may defend itself from (lymph)angiogenesis, the precise role of limbal stem cells situated at the junction linking the vascularized conjunctiva and avascular cornea is still unclear. The way by which the limbal niche and especially the residing stem cell population react to insults such as UV irradiation may provide further insights in understanding the underlying mechanisms which tip the balance towards proangiogenic conditions in the cornea as well as in developing further treatment strategies against inflammation and neovascularisation. Recent research highlights the importance of applying preventative measures against UV irradiation to avoid LESC damage as well as disruption of the limbal barrier leading to conditions such as pterygium. However, as the factors contributing to pterygium pathology are multiple, protection from UV irradiation is not sufficient to prevent recurrence. Therefore, it is important to drive research efforts towards therapies combining surgery with the use of anti-inflammatory and antiangiogenic molecules as well as UV protection (e.g., UV-blocking contact lenses) in order to tackle all risk factors and stop disease progression.
